# Enhancing the reliability of skin tone evaluation: A study of full‐face imaging devices with colour calibration

**DOI:** 10.1111/ics.70090

**Published:** 2026-03-02

**Authors:** Wenyang Wang, Yang Wang, Chanjuan Liu, Andrew Steel, Frédéric Flament

**Affiliations:** ^1^ L'Oréal China Research and Innovation Center Shanghai China

## Abstract

**Background:**

Skin colour measurement is common and essential to clinical evaluation, especially for product efficacy evaluation and research on skin tone and pigmentary disorders. Among multiple evaluation methods, full‐face imaging analysis has become increasingly valuable thanks to attractive visuals, trackability, algorithm optimization, etc. Today, numerous imaging devices are available on the market, employing diffuse light or cross‐polarized light. While most systems control their light source output during manufacturing, lack of precise colour calibration and variations between devices remain the key barriers for product efficacy evaluation for skin tone.

**Objective:**

This research aims to enhance the reliability of colour measurement by validating two representative full‐face imaging devices for clinical objective evaluation. Using a ColorChecker during image capture will allow colour calibration to standardized lighting conditions, thus mitigating device‐specific variations and improving the reliability of skin colour measurements.

**Methods:**

A 4‐week clinical study investigated two imaging devices employing different light sources. A ColorChecker was included in each image capture. Initial analysis of each colour patch assessed the inherent colour accuracy and stability of both devices. Subsequently, image colour calibration was applied to standardize colours to a reference colour under standard light source. Further analysis then evaluated whether this calibration improved the devices' capabilities in measuring skin tone.

**Results:**

There is a disparity in the colour measurement capabilities of the two investigated devices. By incorporating a standard ColorChecker within the field of view and implementing image colour calibration through use of a polynomial regression model, colour accuracy and system stability has been enhanced, thus improving the reliability of colour assessment in clinical evaluation and reducing the inconsistencies between devices.

**Conclusions:**

This study offers insights into colour measurement for skin tone evaluation, demonstrating the value of using a ColorChecker and colour calibration to ensure reliable and accurate skin tone measurements.

## Graphical abstract

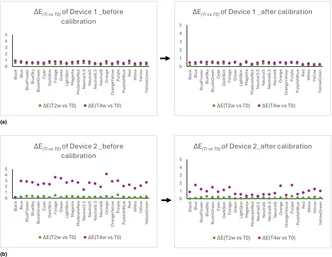

## Résumé

**Contexte:**

La mesure de la couleur de la peau est. fréquente et essentielle pour l'évaluation clinique, en particulier pour l'évaluation de l'efficacité du produit et la recherche sur le teint de la peau et les troubles pigmentaires. Parmi les différentes méthodes d'évaluation, l'analyse d'imagerie sur l'ensemble du visage est. devenue de plus en plus précieuse grâce à des visuels attrayants, la traçabilité, l'optimisation des algorithmes, etc. Aujourd'hui, de nombreux dispositifs d'imagerie sont disponibles sur le marché, utilisant une lumière diffuse ou une lumière polarisée croisée. Bien que la plupart des systèmes contrôlent la sortie de la source lumineuse pendant la fabrication, l'absence d'étalonnage précis des couleurs et les variations entre les dispositifs restent les principaux obstacles à l'évaluation de l'efficacité du produit pour le teint.

**Objectif:**

Cette étude vise à améliorer la fiabilité de la mesure des couleurs en validant deux dispositifs d'imagerie du visage complet représentative pour l'évaluation clinique objective. L'utilisation d'un ColorChecker pendant la capture d'image permettra un étalonnage des couleurs en fonction des conditions d'éclairage normalisées, réduisant ainsi les variations spécifiques au dispositif et améliorant la fiabilité des mesures de la couleur de la peau.

**Méthodes:**

Une étude clinique de quatre semaines a examiné deux dispositifs d'imagerie utilisant différentes sources de lumière. Un ColorChecker était inclus dans chaque capture d'image. L'analyse initiale de chaque patch de couleur a évalué la précision et la stabilité inhérentes des couleurs des deux dispositifs. Ensuite, l'étalonnage des couleurs de l'image a été appliqué pour normaliser les couleurs à une couleur de référence sous une source de lumière standard. Une analyse plus approfondie a ensuite évalué si cet étalonnage améliorait les capacités des dispositifs à mesurer le tonus cutané.

**Résultats:**

Il existe une disparité dans les capacités de mesure des couleurs des deux dispositifs étudiés. En incorporant un ColorChecker standard dans le champ d'exploration et en mettant en œuvre l'étalonnage des couleurs des images grâce à l'utilisation d'un modèle de régression polynomiale, la précision des couleurs et la stabilité du système ont été améliorées, améliorant ainsi la fiabilité de l'évaluation des couleurs dans l'évaluation clinique et réduisant les incohérences entre les dispositifs.

**Conclusions:**

Cette étude offre des informations sur la mesure de la couleur pour l'évaluation du teint de la peau, démontrant l'importance d'utiliser un ColorChecker et de calibrer la couleur pour garantir des mesures fiables et précises du teint de la peau.

## INTRODUCTION

Accurate and reliable skin tone assessment is essential for various applications, from diagnosing pigmentary disorders to evaluating treatment efficacy and developing personalized skincare. While numerous devices measure skin colour, ensuring their accuracy and consistency is paramount.

Imaging skin colour measurement devices typically employ either diffuse or cross‐polarized light, each with distinct advantages. Diffuse light, often generated by integrating spheres, offers a good representation of overall natural colour perception. The images from diffuse light often have softer shadows and evenly distributed light on the face, which reveal surface colour and texture [1, 2]. Cross‐polarized light, created with polarizing filters, effectively eliminates specular reflections to reveal pigment colour. The images from cross‐polarized light often reveal the subsurface scattering, as they have different optical directions than the specular reflections [3, 4]. This study investigates the capabilities of two representative devices—Chromasphere® + (with diffuse light) and VISIA®‐CR 5 (with cross‐polarized light)—for skin tone evaluation.

Colour calibration typically falls into two primary categories: device‐based and image‐based methods. Device‐based methods aim to ensure the device accurately captures or displays colours according to a standardized colour space. Typically, device‐based colour calibration involves using a hardware or software tool, often combined with a standardized colour target like an X‐Rite ColorChecker. The device captures or displays the target, and the software analyses the results to create a device profile. This profile corrects for the device's inherent colour biases, bringing its colour output closer to the standard. On the other hand, image‐based methods serve to correct the colours within a specific image rather than the device itself. It aims to improve the accuracy of colours within a particular scene, often by removing colour casts or adjusting white balance. Both methods play important roles in achieving accurate colour representation, and they can even be used in conjunction. For example, a well‐calibrated device can serve as a solid foundation, minimizing the need for extensive image‐based corrections. In this study, an image‐based method was applied, where a ColorChecker was added within the field of view and software was then used to analyse the ColorChecker's colours within the image to correct for colour inaccuracies. The impact of image calibration on the performance of colour measurement was investigated.

## MATERIALS AND METHODS

### Materials

Two representative imaging devices with different light sources were investigated; a standard ColorChecker was fitted in the field of view (FOV) of the two imaging systems.

- Device 1: Device 1 is the Chromasphere® + (L'OREAL, Paris, France), a spectrophotometer‐based device that employs diffuse illumination for precise skin colour measurements. The original Chromasphere® was specially developed by L'OREAL [5, 6] and utilizes a fibre‐coupled xenon arc lamp as its light source; this device features an integrating sphere with an interior coated in a highly diffusing white paint, thereby ensuring that all colour measurements are conducted under diffuse light. As its successor, the Chromasphere® + retains this diffuse light source feature, but the camera system has been upgraded to a Nikon D750 digital camera. Furthermore, a ColorChecker was added to the field of view to enhance calibration, as demonstrated in the image in Figure 1a.
- Device 2: Device 2 is the VISIA®‐CR (Canfield Scientific Inc., Fairfield, NJ), a multi‐spectral imaging system designed to capture images under various lighting conditions for comprehensive skin analysis. As the latest version of this full‐face imaging system, the VISIA®‐CR 5 is widely utilized across the daily care industry due to its extensive multifunctionality. The device is equipped with advanced LED light sources and utilizes polarizing filters to eliminate specular reflections, which ensures that colour measurements are captured under cross‐polarized light. For the purposes of this study, a ColorChecker was also integrated into the field of view to facilitate precise colour measurement, as illustrated in the image demo in Figure 1b.
- ColorChecker: The X‐Rite ColorChecker® Classic‐Nano, which measures 24 × 40 mm, was fitted within the Field of View (FOV) of both imaging systems. This tool comprises 24 distinct colour patches ranging from natural tones, such as dark skin and light skin, to a variety of chromatic and neutral shades. Specially, the array includes blue sky, foliage, blue flower, bluish green, orange, purplish blue, moderate red, purple, yellow green, orange yellow, blue, green, red, yellow, magenta and cyan. Additionally, it features a greyscale ramp consisting of white, neutral 8, neutral 6.5, neutral 5, neutral 3.5 and black, as illustrated in Figure 1c [7, 8, 9].

**FIGURE 1:**
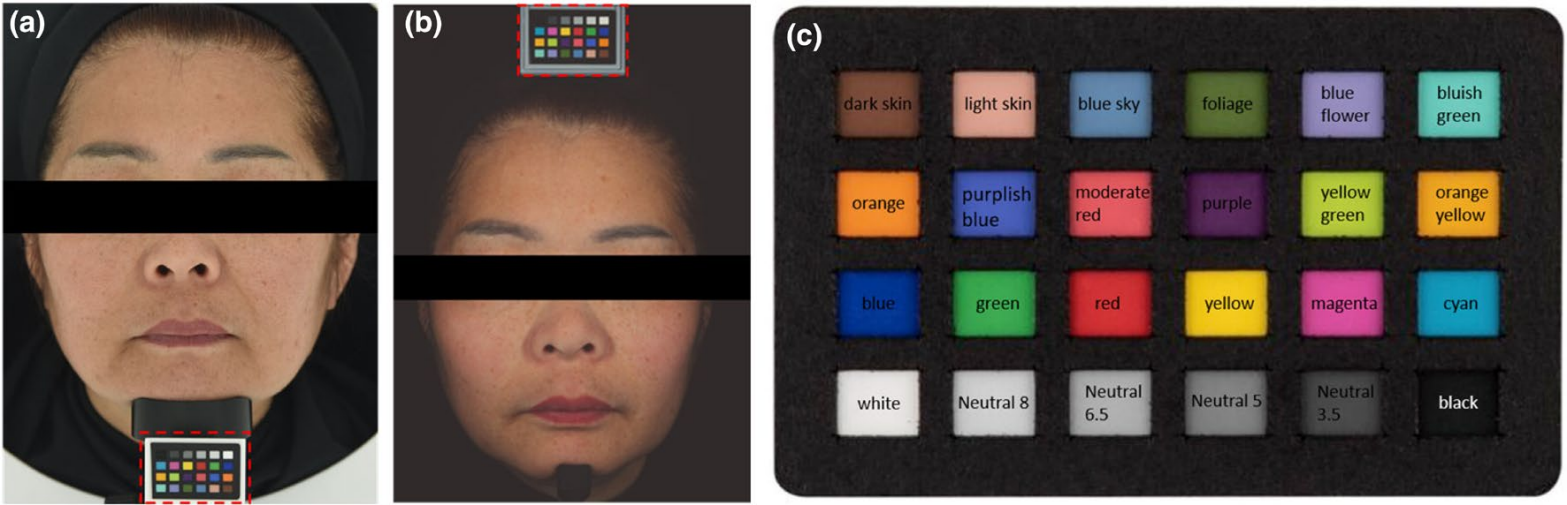
(a) Demo of Chromasphere®+ (b) Demo of VISIA®‐CR 5 (c) X‐Rite® ColorChecker Classic‐Nano was well‐suited for alignment with the field of view (FOV).

### Methods

#### Study design

To mimic the real clinical evaluation, a total of six healthy Chinese female subjects aged from 18 to 60 years old were recruited for participation, all subjects provided informed consent prior to their enrolment in the study and the study protocol was approved by an institutional ethics committee. A typical 4‐week study was conducted, during which three side views (left(L)/right(R)/front(F)) were captured along with six sets of consecutive pictures. Additionally, a consistent site was selected for the ColorChecker to ensure it was always facing the camera, while bi‐weekly measurements were taken using both devices under controlled conditions of 21°C ± 1°C and 50% ± 5% relative humidity. The colour of each colour patch in the ColorChecker in the image was measured and analysed. The colour accuracy was assessed by comparing measured values with known standards, calculating Δ*E*_(measured vs. standard)_ for each patch before and after calibration. While the system stability was evaluated by calculating the Δ*E*_(Ti vs. T0)_ between time points to quantify the consistency of measurements over time.

$$\begin{array}{c} \Delta E_{\left(\text{measured}\;\mathrm{vs}.\;\text{standard}\right)}=\left[{\left({L^{*}}_{\text{measured}}-{L^{*}}_{\text{standard}}\right)}^{2}\right.\\ {\left.+{\left({a^{*}}_{\text{measured}}-{a^{*}}_{\text{standard}}\right)}^{2}+{\left({b^{*}}_{\text{measured}}-{b^{*}}_{\text{standard}}\right)}^{2}\right]}^{1/2} \end{array}$$

$$\Delta E_{\left(\mathrm{Ti}\;\mathrm{vs}.\;\text{T0}\right)}={\left[{\left({L^{*}}_{\mathrm{Ti}}-{L^{*}}_{\text{T0}}\right)}^{2}+{\left({a^{*}}_{\mathrm{Ti}}-{a^{*}}_{\text{T0}}\right)}^{2}+{\left({b^{*}}_{\mathrm{Ti}}-{b^{*}}_{\text{T0}}\right)}^{2}\right]}^{1/2}$$

#### Colour calibration

The colour calibration was performed for each of the collected images, the details of the calibration method are as follows:

A ColorChecker of known colorimetric values under standard lighting conditions was included within the field of view for both devices as the calibration reference. For each acquired image, the measured RGB values of every ColorChecker patch were extracted. Let

$$m_{i}={\left[R_{i}^{\left(m\right)},G_{i}^{\left(m\right)},B_{i}^{\left(m\right)}\right]}^{⊤}$$

denote the measured RGB vector for the *i*‐th patch, and

$$r_{i}={\left[R_{i}^{\left(r\right)},G_{i}^{\left(r\right)},B_{i}^{\left(r\right)}\right]}^{⊤}$$

represent the corresponding reference RGB value provided by the manufacturer under D65 illumination.

To minimize the discrepancy between measured and reference colour values, a polynomial regression model was derived for each image to transform the acquired image colour space into the standard reference colour space. In the general form, the calibration model can be expressed as follows:

$$r_{i}=W\;\phi \left(m_{i}\right),$$

where $\phi \left(m_{i}\right)$ is the polynomial feature expansion of the measured RGB values, and W is the regression coefficient matrix. A second‐order polynomial model was implemented in our study, which expands the RGB vector as

$$\begin{array}{c} \phi \left(m_{i}\right)=\left[1,\;R_{i}^{\left(m\right)},\;G_{i}^{\left(m\right)},\;B_{i}^{\left(m\right)},\;{R_{i}^{\left(m\right)}}^{2},\;{G_{i}^{\left(m\right)}}^{2},\;{B_{i}^{\left(m\right)}}^{2}\right.\\ {\left.\;R_{i}^{\left(m\right)}G_{i}^{\left(m\right)},\;R_{i}^{\left(m\right)}B_{i}^{\left(m\right)},\;G_{i}^{\left(m\right)}B_{i}^{\left(m\right)}\right]}^{⊤}. \end{array}$$

For all N ColorChecker patches in a given image, the calibration model can be written compactly in matrix form. Let

$$R=\left[\begin{array}{c} r_{1}^{⊤}\\ r_{2}^{⊤}\\ \vdots \\ r_{N}^{⊤} \end{array}\right]\in {\mathbb{R}}^{N\times 3}$$

denote the matrix of reference RGB values, and

$$\Phi =\left[\begin{array}{c} \phi {\left(m_{1}\right)}^{⊤}\\ \phi {\left(m_{2}\right)}^{⊤}\\ \vdots \\ \phi {\left(m_{N}\right)}^{⊤} \end{array}\right]\in {\mathbb{R}}^{N\times K}$$

the corresponding design matrix of polynomial features evaluated at each measured RGB vector $m_{i}$, where K is the number of polynomial terms. The regression coefficient matrix is defined as

$$W\in {\mathbb{R}}^{3\times K},$$

so that the model for all patches becomes

$$R\approx \Phi \;W^{⊤}.$$

The optimal calibration matrix W is obtained by solving the least‐squares problem

$$W^{⊤}=\arg\min_{A\in {\mathbb{R}}^{K\times 3}}{∥R-\Phi \;A∥}_{F}^{2},$$

which admits the closed‐form solution

$$W^{⊤}={\left(\Phi^{⊤}\Phi \right)}^{-1}\Phi^{⊤}R,$$

assuming $\Phi^{⊤}\Phi$ is invertible.

Once the transformation matrix W is computed, the calibrated RGB value for any pixel in the acquired image is obtained by

$$p^{\left(c\right)}=W\;\phi \left(p^{\left(m\right)}\right),$$

where $p^{\left(m\right)}$ is the original measured RGB value and $p^{\left(c\right)}$ is the corrected value in the reference colour space. This calibration process was developed and implemented following the equations above and the flow chart below using MATLAB 2022b (Figure 2).

**FIGURE 2:**
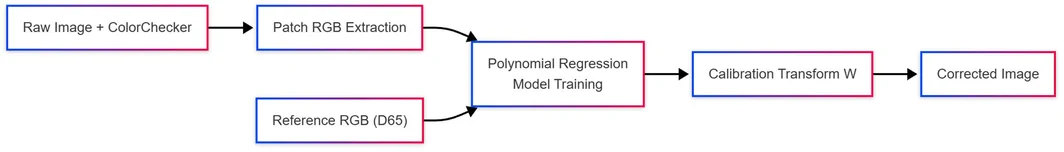
Flow chart of colour calibration.

### Statistical analysis

Data collected from all images were used for statistical analysis. Student's *t*‐test was used to compare mean values between any two groups, and values of *p* < 0.05 were considered statistically significant. Statistical analyses were performed using SAS software version 9.4 (SAS Statistical Institute, Cary, USA).

## RESULTS

Image analysis on CIE L*a*b* system is a widely used tool for product efficacy on skin tone benefit. Colour accuracy is essential for obtaining reliable evaluation results, especially when performing absolute value measurements, as it directly determines the validity of the data and the scientific basis of product claims. Concurrently, system stability is equally critical, ensuring consistent representation of skin colour over time, preventing measurement deviations and thus guaranteeing the accuracy and efficiency of long‐term evaluations.

### Before colour calibration

#### Colour accuracy

D65 is a standard illuminant defined by the International Commission on Illumination (CIE), its spectral power distribution closely matches the average midday daylight, including ultraviolet components, it is used to simulate daylight conditions, ensuring that colours appear as they would under natural daylight. In this study, the standard values of ColorCheker under D65 light source is used as a reference. Both devices exhibit limitations potentially stemming from their light source settings, including colour temperature and illumination intensity, etc. Device 1 demonstrates superior colour accuracy compared to Device 2, as evidenced by its lower mean Δ*E*_(measured vs. standard)_ values (Figure 3), with a significant difference (*p* < 0.05) between devices. This indicates a smaller discrepancy between the measured colour and the reference colour for Device 1. In perceptually uniform colour spaces like CIE L*a*b*, where numerical changes correspond to perceived colour differences, balanced representation is key for visual quality and consistency. The less dispersed Δ*E*_(measured vs. standard)_ distribution across the 24 colour patches for Device 1 (Figure 3) further suggests its superior colour space balance.

**FIGURE 3:**
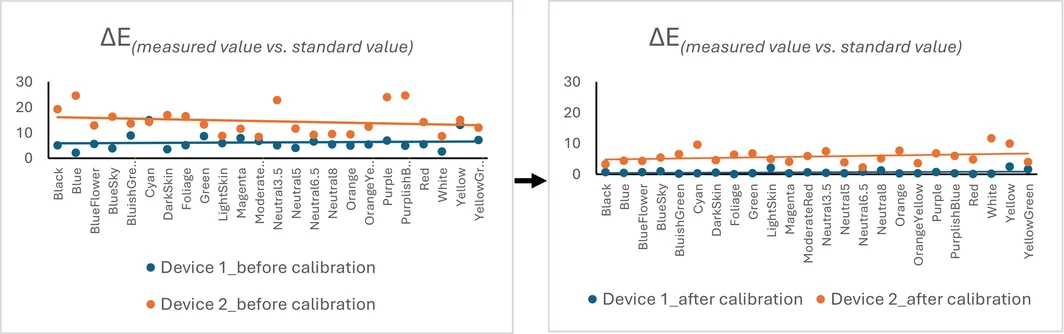
Colour difference (ΔE) between measured value and known standard value (under D65) of ColorChecker (24 colour patches) before (Left) & after (Right) calibration: (1) the closer the value is to zero, the better the accuracy; (2) the smaller the data dispersion of 24 colour patches, the better the colour space balance.

#### System stability

As Figure 4 shows, Device 2 shows good stability at T2w, but at T4w, it exhibits a mean Δ*E*_(T4w vs. T0)_ of approximately 2.50, a visually perceptible colour difference [10], and there was a significant difference (*p* < 0.05) between T2w and T4w. This instability could compromise the reliability of real skin tone evaluation data, making it difficult to discern whether observed changes result from treatment or device fluctuations. The hypothesis of Device 2's instability may relate to the flash mode in the operation, although the LED light source does not typically experience significant attenuation, they may not achieve a steady state instantaneously. In contrast, Device 1, with a mean Δ*E*_(T4w vs. T0)_ of about 0.67, demonstrates better stability and there was no significant difference between time points. This is likely attributed to its use of xenon lamp strips, which are less prone to fluctuation once a steady state is reached, and its continuous ‘on’ mode of operation rather than a flashing mode. Therefore, Device 1 exhibits superior system stability compared to Device 2.

**FIGURE 4:**
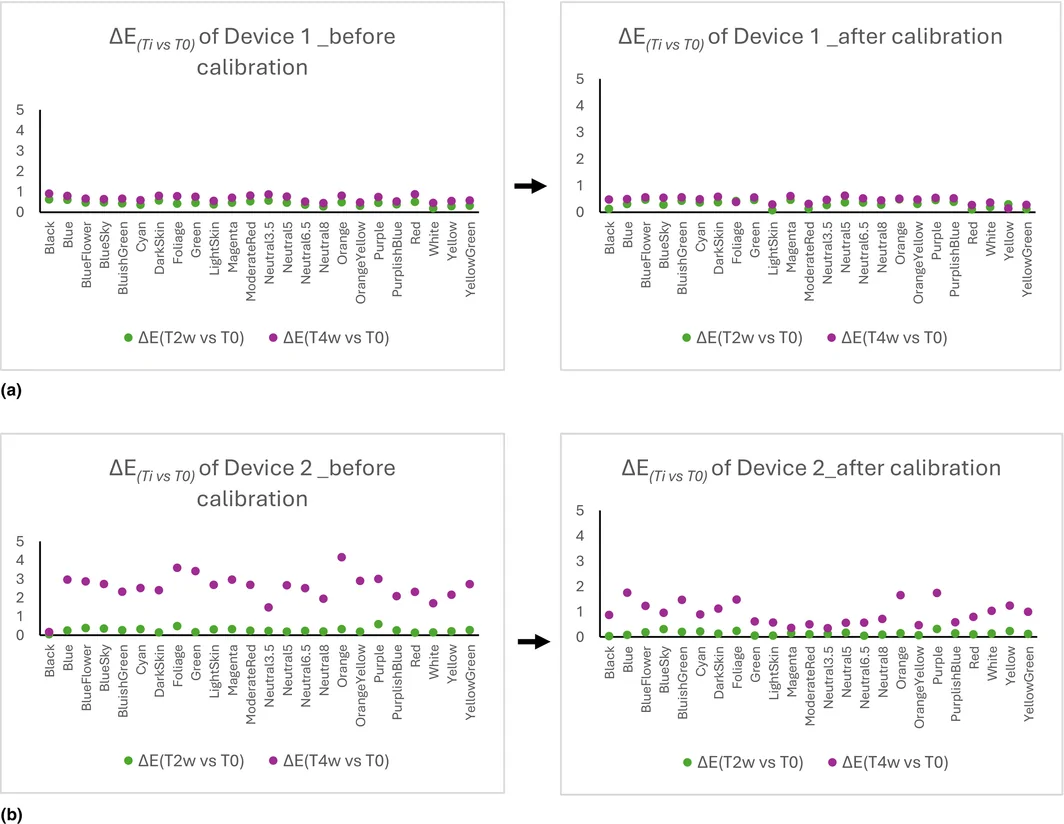
Colour difference (ΔE) between time points of ColorChecker (24 colour patches) before (Left) and after (Right) calibration. (a) Device 1 (b) Device 2: The closer the value is to zero, the better the system stability.

### After colour calibration

Image‐based colour calibration method was performed using a polynomial regression model; colour calibration matrices were derived for each image, transforming the acquired image colour space to the standard colour space defined by the ColorChecker.

#### Colour accuracy

The calibration demonstrably improved colour accuracy for both devices, reducing the difference between measured and standard values for the 24 colour patches and decreasing data dispersion. Post‐calibration, Device 1 consistently outperformed Device 2 with lower mean Δ*E*_(measured vs. standard)_ values and less dispersion, confirming its superior colour accuracy. The device's inherent capability serves as the foundation; a solid foundation can minimize the need for extensive image‐based corrections, and the images taken will be easier to calibrate.

#### System stability

The calibration also significantly enhanced system stability. For Device 2, the mean Δ*E*_(T4w vs. T0)_ significantly decreased from 2.50 to 0.93, effectively mitigating its system limitations and the ΔE between time points at T2w was even smaller. Device 1's already strong stability was further improved, with a post‐calibration colour difference between time points of less than 0.5.

While both devices benefited, the improvement was more pronounced for Device 2, likely because Device 1's inherently better performance required fewer image‐based corrections. This image‐based colour calibration method serves as a valuable complement to device‐based calibration, significantly enhancing colour measurement accuracy and stability.

## DISCUSSION

The main objective of this study was to enhance the reliability of colour measurement for skin tone evaluation; this study effectively employed a computational approach to mitigate colour variation issues associated with the difference of device, light source, imaging system, imaging condition, etc. In the pursuit of accurate colour assessment for skin tone, the key is the calibration process; it was applied using MATLAB 2022b platform to derive a polynomial regression model. Our research demonstrates that while Device 1 offers inherently better colour accuracy and stability in skin tone evaluation due to its advanced technology, colour calibration using a ColorChecker proves crucial for optimizing the performance of both devices. Specifically, for Device 2, this calibration creates an image‐specific profile that mitigates its inherent limitations, improving colour accuracy and consistency to approach the level of Device 1. This underscores the value of image‐based colour calibration as a method to improve the reliability and consistency of skin tone assessments regardless of the imaging system used. On the other hand, the inherent performance of the device itself is the foundation; the method for colour accuracy and system stability validation might also inspire the device‐based calibration, which might reduce the needs of image corrections. In recent years, AI‐based colour calibration has also been gaining momentum, with the strength to handle complex scenes and lighting conditions more effectively, potential for higher accuracy and more natural‐looking results. However, it requires a large database for training. Some AI models can make it difficult to understand why specific adjustments are made. Computational resources may be a concern, especially for high‐resolution images or real‐time applications. The potential biases present in the training data may affect the results. Adoption in professional workflows is still ongoing.

## CONCLUSION

This study provides valuable insights for researchers and clinicians engaged in the practice of skin tone evaluation. While our findings indicate that the Chromasphere® + possesses inherently greater accuracy and stability in its measurements compared to another device included in this investigation, the study strongly emphasizes the crucial importance of incorporating a standardized colour calibration method, irrespective of the specific device employed. This practice is essential for achieving the most reliable and comparable skin tone assessments across different devices, studies and time points. Standardized calibration minimizes variability introduced by image‐specific characteristics, environmental factors and temporal drift, thereby ensuring consistent and trustworthy data for both research and clinical applications. By adhering to a standardized colour calibration protocol, researchers and clinicians can improve the validity and comparability of their skin tone assessments, leading to more robust conclusions and more effective clinical decisions.

## AUTHOR CONTRIBUTIONS

W.W. and C.L. performed the research. W.W. and Y.W. designed the research study. C.L. contributed to image analysis and calibration methodology. W.W. wrote the original manuscript. Y.W. and C.L. reviewed and edited the manuscript. A.S. and F.F. contributed to supervision and project administration. All authors have read and approved the final manuscript.

## CONFLICT OF INTEREST STATEMENT

The authors declare no conflicts of interest.

## Data Availability

The data that support the findings of this study are available on request from the corresponding author. The data are not publicly available due to privacy or ethical restrictions.
